# Association between vitamin D insufficiency and tuberculosis in a vietnamese population

**DOI:** 10.1186/1471-2334-10-306

**Published:** 2010-10-25

**Authors:** Lan T Ho-Pham, Nguyen D Nguyen, Tong T Nguyen, Dung H Nguyen, Phuong K Bui, Vien N Nguyen, Tuan V Nguyen

**Affiliations:** 1 Department of Internal Medicine, Pham Ngoc Thach University of Medicine, Thanh Thai Street, District 10, Ho Chi Minh City, Vietnam; 2 People's Hospital 115, Thanh Thai Street, District 10, Ho Chi Minh City, Vietnam; 3 MEDIC Medical Center, 254 Hoa Hao Street, District 10, Ho Chi Minh City, Vietnam; 4 Pham Ngoc Thach Hospital for Tuberculosis, 120 Hong Bang Street, District 5, Ho Chi Minh City, Vietnam; 5 Osteoporosis and Bone Biology Program, Garvan Institute of Medical Research, 384 Victoria Street, Sydney, NSW 2010, Australia; 6 School of Public Health and Community Medicine, University of New South Wales, Sydney, NSW 2052, Australia; 7 St Vincent's Clinical School, University of New South Wales, Victoria Street, Sydney, NSW 2010, Australia

## Abstract

**Background:**

Recent *in vitro *evidence suggests a link between vitamin D status and the risk of tuberculosis (TB). This study sought to examine the association between vitamin D status, parathyroid hormone (PTH) and the risk of TB in a Vietnamese population.

**Methods:**

The study was designed as a matched case-control study, which involved 166 TB patients (113 men and 53 women), who were age-and-sex matched with 219 controls (113 men and 106 women). The average age of men and women was 49 and 50, respectively. TB was diagnosed by the presence of acid-fast bacilli on smears from sputum, and the isolation of *M. tuberculosis*. All patients were hospitalized for treatment in a TB specialist hospital. Controls were randomly drawn from the general community within the Ho Chi Minh, Vietnam. 25-hydroxyvitamin D [25(OH)D] and PTH was measured prior to treatment by an electrochemiluminescence immunoassay (ECLIA) on a Roche Elecsys. A serum level of 25(OH)D below 30 ng/mL was deemed to be vitamin D insufficient.

**Results:**

The prevalence of vitamin D insufficiency was 35.4% in men with TB and 19.5% in controls (*P *= 0.01). In women, there were no significant differences in serum 25(OH)D and serum PTH levels between TB patients and controls. The prevalence of vitamin D insufficiency in women with TB (45.3%) was not significantly different from those without TB (47.6%; *P *= 0.91). However, in both genders, serum calcium levels in TB patients were significantly lower than in non-TB individuals. Smoking (odds ratio [OR] 1.25; 95% confidence interval [CI] 1.10 - 14.7), reduced 25(OH)D (OR per standard deviation [SD]: 1.14; 95% CI 1.07 - 10.7) and increased PTH (OR per SD 1.13; 95% CI 1.05 - 10.4) were independently associated with increased risk of TB in men.

**Conclusion:**

These results suggest that vitamin D insufficiency was a risk factor for tuberculosis in men, but not in women. However, it remains to be established whether the association is a causal relationship.

## Background

Contrary to popular belief, tuberculosis (TB) is still an on-going and important public health problem in the world. In the year 2008 alone, there were 9.4 million new cases of TB worldwide [1]. Moreover, according to the World Health Organization, at present, one-third of the world's population is infected with TB; of which, 34% are from Southeast Asian countries [2]. In Vietnam, a Southeast Asian country, TB has historically been a public health concern, due to a high prevalence and incidence in the general population. Recent statistics indicated that during 1997-2004 there were ~48,500 cases of TB (or 70 per 100,000 population), and the incidence increased by around 0.2% per year, which occurred mainly in rural areas [3]. However, it is not clear why the incidence of TB in Vietnam has been on the rise in recent years.

In recent years, several lines of evidence suggest a link between vitamin D and TB [4-7]. The active form of vitamin D (1,25(OH)2D3) has been shown to inhibit growth of Mycobacterium tuberculosis (*M. tuberculosis*) through stimulating cell-mediated immunity and activating monocytes [6]. Studies in Gujarati Asians living in the UK found that lower levels of 25(OH)D were associated with an increased risk of pulmonary TB [8]. However, a study in Indonesia found no significant difference in 25(OH)D levels between TB and non-TB individuals [9]. Nonetheless, a recent systematic review and meta-analysis suggested that individuals with TB had lower levels of 25(OH)D than non-TB individuals [10]. The conclusion is, however, not definite because the association could be confounded by lifestyle factors and sunlight exposure which were not considered in the analysis.

While the association between vitamin D and TB has been studied in many Asian communities in Western countries, few studies have been done in Asian populations. In Vietnam, a tropical country, we have recently shown that the prevalence of vitamin D insufficiency in Vietnam is high [11]. Given the biologic link between vitamin D and immunity, we hypothesized that TB patients had lower serum levels of vitamin D than healthy individuals. This study was designed to test the hypothesis by examining vitamin D status and parathyroid hormone in the Vietnamese population.

## Methods

### Study design and subjects

The study setting was Ho Chi Minh City, Vietnam. The City is located at 10°45'N, 106°40'E in the southeastern region of Vietnam. The City, being close to the sea, has a tropical climate, with an average humidity of 75%. There are only two distinct seasons: the rainy season, with an average rainfall of about 1,800 millimetres annually (about 150 rainy days per year), usually begins in May and ends in late November; the dry season lasts from December to April. The average temperature is 28°C (82°F), the highest temperature sometimes reaches 39°C (102°F) around noon in late April, while the lowest may fall below 16°C (61°F) in the early mornings of late December. The present study was taken place between April and May 2009.

The research protocol and procedures were approved by the Medical Research Committees of the People's Hospital 115 and Pham Ngoc Thach University of Medicine. All participants were provided with full information about the study's purpose and gave informed consent to participate in the study, according to the principles of medical ethics of the World Health Organization.

The study was designed as a case-control investigation, in which each case of TB was matched with 1 (for men) or 2 controls (for women), with the matching variables being age and sex. The matching ratio 1:2 for women was done to ensure the adequate of sample size for detecting an association between vitamin D and TB. The tolerance limit of age was 1 year. We used the "greedy matching algorithm" (which has been implemented in a SAS macro by Mayo Clinic) for matching data [12]. The cases were recruited from Pham Ngoc Thach Hospital (Ho Chi Minh City), which is a tertiary teaching hospital specializing in TB treatment. The data collection was undertaken as part of the National Tuberculosis Program. During the period, all patients admitted to the Hospital and diagnosed with TB, prior to commencement of anti-TB treatment, were invited to participate in the study.

TB was ascertained according to a standard procedure, in which there was a presence of acid-fast bacilli (AFB) on smears from sputum, and the isolation of *M. tuberculosis *on culture. A definitive diagnosis of TB was made when patients met one of following criteria: (a) at least two positive AFB smears from two different sputum smears; (b) one positive AFB smear and positive culture; and (c) one positive AFB smear and typical result of lung TB infection on chest X-ray. AFB was assessed using either fluorescence microscopy (auramine-rhodamine staining) or Ziehl-Neelsen staining method. Sputum must contain 5,000 to 10,000 bacilli/mL to be considered positive.

The control group was randomly recruited from various districts within the Ho Chi Minh City from another study on vitamin D status in an urban population in Vietnam, in which study procedures have been described elsewhere [11]. Briefly, we approached community organizations, including churches and temples, and obtained the list of members, and then randomly selected individuals aged 18 or above. We sent a letter of invitation to the selected individuals. Participants were excluded from the study if they had diseases deemed to affect to vitamin D metabolism such as malabsorption syndrome, renal failure. In addition, individuals with prolonged immobility (over 2 months) were not recruited to the study. The recruitment of cases and controls was taken place at approximately the same time.

### Measurement of vitamin D

Prior to commencing TB treatment, fasting serum was obtained for total calcium, creatinine, liver enzymes, and parathyroid hormone (PTH) and 25-hydroxyvitamin D [25(OH)D]. Concentration of 25(OH)D and PTH in serum was measured by electrochemiluminescence immunoassay (ECLIA) on a Roche Elecsys 10100/201 system (Roche Diagnosis Elecsys). This method can measure the concentration of 25(OH)D in the range of 4-100 ng/ml (10-250 nmol/L), and PTH in the range 1.2-500 pg/ml (0.127-530 pmol/L). The sensitivity of the assay is 1.5 ng/ml with an intraassay CV of 5.6% at 15.9 ng/ml and 11.6% at 58.9 ng/ml. The inter-assay CV at these two levels was 9 and ~12%, respectively.

### Data collection

A questionnaire relating to anthropometry, clinical history, lifestyle, physical activity, dietary habit, fracture and falls, were developed and used in the data collection. The questionnaire collected anthropometric data such as age, height and weight. Age was calculated from the date of birth to the date of interview. Height without shoes (in centimeters) was measured to the nearest 0.1 cm by a wall-mounted stadiometer. Weight, without shoes or clothing, was measured (to the nearest 0.1 kg) on an electronic scale. Body mass index (BMI) was then derived as the ratio of weight (kg) over height squared (in m^2^).

Each participant was asked to provide information on current and past smoking habits. This was quantified in terms of the number of pack-years consumed in each ten-year interval age group. Alcohol intake in average numbers of standard drinks per day, present as well as within the last 5 years, was obtained.

### Statistical analysis

Circulating vitamin D levels were classified into four groups according to the following criteria: those undetectable (< 15 ng/mL), those deficient (< 20 ng/mL), those insufficient (< 30 ng/mL), and those sufficient (≥ 30 ng/mL). The primary purpose of analysis was to assess the association between vitamin D status and the risk of TB. The main statistical model was the logistic regression analysis, in which TB status was considered the primary outcome. Circulating vitamin D level was treated as a predictor. Clinical factors and lifestyle factors were considered as covariates. The association between 25(OH)D and TB was expressed as odds ratio and 95% confidence interval. The R program was used for the statistical analysis [13].

## Results

The study included 166 patients (113 men and 53 women) with TB, and 219 controls (113 men and 106 women). The average age of men and women was 49 and 50, respectively. By design, there was no significant difference in age between TB and controls. As expected, men and women with TB had lower body weight than controls. About 78% of men with TB were smokers, and this prevalence was significantly higher than in controls (61%, *P *= 0.007). However, in women, there was no significant difference in the prevalence of smoking between TB patients and controls (*P *= 0.99). In women, the prevalence of alcohol use was 7.5%, which was significantly higher than in controls (*P *= 0.01). In men, although the prevalence of alcohol use in TB patients was higher than in those without TB (64% vs 59%), the difference was not statistically significant (*P *= 0.44).

In men, serum levels of 25(OH)D were lower (by 10%) and serum levels of PTH were higher (by 20%) in patients with TB than in controls. In women, there were no significant differences in 25(OH)D and PTH between TB patients and controls. However, in both genders, serum calcium levels in TB patients were significantly lower than in non-TB individuals (Table 1).

**Table 1:** Clinical and demographic characteristics of study participants

Variable	Controls	Tuberculosis	MD (95% CI)	P-value
**Women**				
N	106	53		
Age (yr)	49(20)	50(20)	-0.7(-7.4, 5.9)	0.831
Height (cm)	153(6)	154(4)	-1.4(-3.1, 0.4)	0.118
Weight (kg)	51(7)	43(6)	7.7(5.4, 10.0)	< .001
Log[25(OH)D, ng/mL]	3.39(0.18)	3.40(0.24)	-0.01(-0.08, 0.05)	0.706
Log[PTH, pg/mL]	3.47(0.39)	3.40(0.65)	0.07(-0.10, 0.23)	0.430
Serum calcium	2.35(0.26)	2.11(0.38)	0.24(0.14, 0.35)	< .001
Use of corticosteroid (n; %)	14(13.21)	6(11.32)		0.735
Use of alcohol (n; %)	0	4(7.55)		0.011
Ever smoking (n; %)	1(0.94)	1(1.89)		1.000

**Men**				
N	113	113		
Age (yr)	49(16)	50(20)	-0.1(-4.2, 4.0)	0.949
Height (cm)	163(6)	154(4)	-2.1(-3.6, -0.5)	0.009
Weight (kg)	61(10)	43(6)	13.1(10.7, 15.5)	< .001
Log[25(OH)D, ng/mL]	3.60(0.26)	3.49(0.24)	0.10(0.04, 0.17)	0.003
Log[PTH, pg/mL]	3.38(0.38)	3.40(0.65)	0.23(0.11, 0.35)	< 0.001
Serum calcium	2.34(0.30)	2.11(0.38)	0.28(0.19, 0.36)	< .001
Use of corticosteroid (n; %)	7(6.19)	9(8.04)		0.591
Use of alcohol (n; %)	67(59.29)	72(64.29)		0.441
Ever smoking (n; %)	69(61.06)	87(77.68)		0.007

Values are mean (standard deviation), unless otherwise specified

MD, mean difference

**Figure 1:**
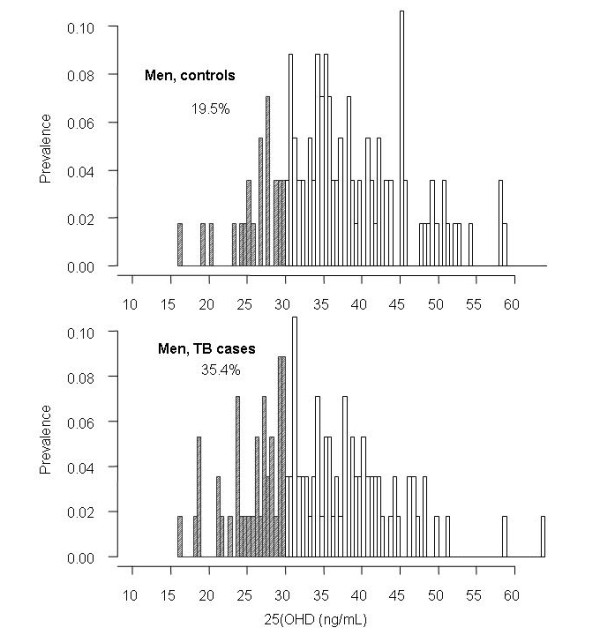
**Prevalence of vitamin D insufficiency (25(OH)D < 30 ng/mL) in controls (upper panel) and in cases with tuberculosis (lower panel), men**.

**Figure 2:**
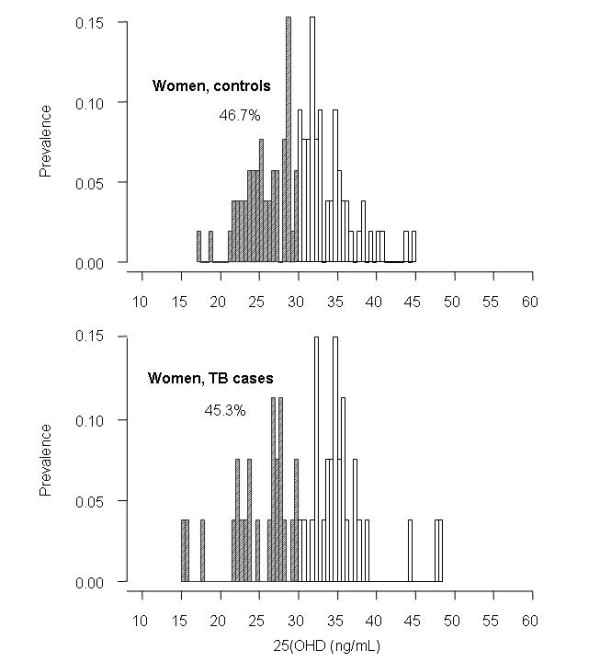
**Prevalence of vitamin D insufficiency (25(OH)D < 30 ng/mL) in controls (upper panel) and in cases with tuberculosis (lower panel), women**.

Using the criteria of 25(OH)D < 30 ng/mL, the prevalence of vitamin D insufficiency was 35.4% in men with TB, and this prevalence was significantly higher than controls (19.5%, *P *= 0.01; Figure 1). Using the criteria of 25(OH)D < 20 ng/mL, the prevalence of vitamin D deficiency was 13.3% in men with TB, > 2-fold higher than in men without TB (5.3%, *P *= 0.07). However, in women, there was no significant difference in vitamin D insufficiency (45% in TB vs 48% in non-TB; Figure 2) or vitamin deficiency (21% vs 19%) between TB patients and non-TB individuals. (Table 2).

**Table 2:** Prevalence of hypovitaminosis D in tuberculosis and control by various 25(OH)D cut-off values

25(OHD) levels (ng/mL)	Controls	Tuberculosis	P-value
Women (n)	106	53	
< 20	2(1.9)	3(5.7)	0.428
< 25	20(19.1)	11(20.8)	0.966
< 30	50(47.6)	24(45.3)	0.913
< 50	(100.0)	(100.0)	

Men (n)	113	113	
< 20	2(1.8)	5(4.4)	0.442
< 25	6(5.3)	15(13.3)	0.067
< 30	22(19.5)	40(35.4)	0.011
< 50	102(90.3)	110(97.4)	0.053

Number in bracket is percentage of group-specific total.

In univariate logistic regression analysis, lower body weight and lower serum levels of calcium were each associated with increased risk of TB in women. However, in men, several risk factors were identified: smoking, reduced height, weight, 25(OH)D, and calcium, and increased PTH (Table 3). In the multiple logistic regression model, smoking, reduced 25(OH)D and increased PTH were independently associated with increased risk of TB in men. None of these factors was associated with TB risk in women (Table 4).

**Table 3:** Risk factors for tuberculosis, univariate analysis

	Unit of comparison	Women	Men
		
		OR (95% CI)	OR (95% CI)
Age (y)*	+10	1.00(0.97, 7.19)	1.00(0.96, 7.13)
Height (cm)	+5	1.06(0.99, 8.41)	**1.07(1.02, 8.84)**
Weight	-5	**1.15(1.10, 11.00)**	**1.14(1.11, 10.59)**
25(OH)D (ng/mL)	-5	0.98(0.92, 6.61)	**1.10(1.03, 9.56)**
PTH (ng/L)	-15	0.98(0.91, 6.60)	**1.11(1.03, 9.81)**
Serum calcium	-0.3	**1.16(1.09, 11.36)**	**1.20(1.14, 12.61)**
Corticosteroid use	yes	0.96(0.77, 6.35)	1.07(0.83, 8.77)
Alcohol use	yes	1.98(1.25, 96.32)	1.05(0.92, 8.33)
Smoking	yes	1.18(0.61, 12.05)	**1.22(1.06, 13.17)**

* Cases and controls were matched by age

**Table 4:** Risk factors for tuberculosis, multivariable analysis

	Unit of comparison	Women	Men
		
		OR (95% CI)	OR (95% CI)
**Model 1**			
Weight (kg)	-5	1.15(1.11, 11.08)	**1.13(1.10, 10.3)**
25(OH)D (ng/mL)	-1SD*	0.98(0.93, 6.74)	**1.07(1.01, 8.52)**
PTH (pg/mL)	-15	0.96(0.90, 6.30)	**1.10(1.03, 9.53)**
Smoking	yes	1.15(0.62, 10.97)	**1.16(1.04, 11.40)**

**Model 2**			
25(OH)D (ng/mL)	-1SD*	0.97(0.91, 6.55)	**1.14(1.07, 10.70)**
PTH (pg/mL)	-15	0.97(0.91, 6.58)	**1.13(1.05, 10.42)**
Smoking	yes	1.08(0.54, 9.10)	**1.25(1.10, 14.67)**

*1SD (ng/mL), 5 in women and 10 in men

## Discussion

Although several lines of *in-vitro *evidence suggest a link between vitamin D and immunity, the *in-vivo *association between vitamin D status and tuberculosis is still a contentious issue. In this study we found that vitamin D insufficiency, as assessed by 25(OHD), was a risk factor for TB in men, but not in women. This finding adds to the growing evidence that vitamin D plays a role in the regulation of *M. tuberculosis*.

The possible association between vitamin D and tuberculosis was first reported more than 20 years ago [14], but subsequent studies have yielded conflicting findings. A number of studies in Gujarati Indian [8], Indian, Pakistani, Somali, Afghan, Sri Lankan and African residents in London [15], and African immigrants living in Australia [16] all have shown that tuberculosis had lower levels of 25(OH)D and higher prevalence of vitamin D deficiency than non-TB individuals. Other studies in Kenya [17] and West Africa [18] have also reported that TB patients had lower 25(OH)D levels compared to non-TB individuals. However, there was no significant difference in 25(OH)D levels between TB patients and controls in Indonesia [9] and Hong Kong [19]. Nevertheless, a recent meta-analysis concluded that TB patients had about 0.70 standard deviation (95% CI 0.42, 0.93) lower 25(OH)D concentration than non-TB individuals [10]. In our study the effect size (in men) was 0.42, which was in the lower range of the effect size found in the meta-analysis. Thus, overall, our finding is broadly consistent with most previous studies.

The observation that PTH was raised in TB patients is probably not surprising, given the inverse relationship between PTH and 25(OH)D [20,21]. However, in this study we found that both reduced 25(OH)D levels and increased PTH levels were independently associated with an increased risk of TB, suggest that PTH may have prognostic value for the assessment of TB risk. We also found that in both genders, serum levels of calcium was lower in TB patients than in controls. However, it should be noted that in our study we did not measure albumin, and uncorrected-for-albumin calcium levels could be driven by albumin levels which are known to be lower in TB patients. A previous study in African population suggested that TB patients had higher serum levels of calcium [17]. This is again consistent with the physiologic understanding that the vitamin D hormone system and parathyroid hormone are the principal regulators of serum concentrations of calcium [21]. However, the finding raises the question that TB patients may be at risk of increased bone loss and osteoporosis.

The observation of low 25(OH)D levels in TB patients could be due to nutritional factors, however, this is unlikely. It is well-known that most (90%) of vitamin D is synthesized in the skin under the influence of ultraviolet sunlight of the sun, and only 10% of vitamin D is obtained from food, mainly salmon, cod fish, and dairy products. In this study, we did not assess dietary calcium intake, but typical Vietnamese diets in general contain virtually very little salt water fishes (such as cod fish and salmon) and little dairy and thus have low calcium [22]. In a previous population based study, we have shown that approximately 45% women and 20% men were vitamin D insufficient. Thus, nutritional factors are unlikely to cause the high prevalence of low vitamin D in TB patients.

While low 25(OH)D and calcium levels in TB patients could be a consequence of the disease, it is highly possible that vitamin D is an antecedent risk factors for TB. The relationship between vitamin D and tuberculosis may be mediated through two mechanisms: increased production of cathelicidin and enhancement of macrophage ability. 1,25(OH)2D, the active form of vitamin D, may enhance the production of LL-37, one of the class of defensins-antimicrobial peptides of the cathelicidin family, culminating in TB destruction [6,23]. Furthermore, vitamin D also restricts intracellular growth of TB via enhancement of macrophage ability. Avoidance of phago-lysosome fusion within the macrophage constitutes a key survival mechanism of TB. The ability of mycobacteria to hinder phagosomal progression, a condition known as phagosome maturation arrest [24], is partly achieved via its retention of the host's tryptophanaspartate-containing coat protein (TACO/coronin-1) [25]. Taken together, these data seem to suggest that low vitamin D status is an antecedent risk factor for TB.

The sex-specific association between vitamin D and TB is a notable finding, which deserves some remarks. We found the association between vitamin D status and the risk of TB in men, not in women. This finding is actually consistent with a previous study in West African population, in which the vitamin D - TB association was only observed in men, not in women [18]. In that study [18], women with TB appeared to have *lower *prevalence of vitamin D insufficiency than women without TB (38% vs 41%). The underlying reason for this sex-specific association is not clear. However, men in general have higher risk of tuberculosis than women [26], and this has been attributed to the sex-related differences in social economic and health care access [26] which predispose men to a greater exposure to TB than women. Recently, it has been proposed that estrogen is a potential mediator that could account for the lower risk of TB in women [27]; however, this hypothesis remains to be tested in empirical studies.

The present study represents one of the largest studies of vitamin D status and TB patients in Asian populations. The study was a matched case control design, and as such, it increased the reliability of estimates of effect size. The study population was highly homogeneous, which reduced the effects of potential ethnic confounders that could compromise the estimates. Moreover, the technique of measurement of 25(OH)D was a novel Elecsys Vitamin D3 automated assay, which has been shown to be a precise method for measuring vitamin D over a wide reportable range in serum. Indeed, recent studies have shown that measurement of 25(OH)D by this method was highly concordant with the HPLC and liquid chromatography tandem mass spectrometry methods [28]. Nevertheless, the study has a number of potential weaknesses. Because the study was a case-control design, no causal inferences could be made for the observed association between vitamin D levels and TB. We could not measure vitamin D2 (ergocalciferol) and 1,25D in this study; however, the occurrence of this vitamin D (~10% of sera [29]) seems not to be a major problem. We did not ascertain the time of exposure to sunlight among cases and controls. However, in this study most TB patients were from rural areas, and rural men normally have higher levels of sun exposure than women. It could, therefore, be argued that the data represented an underestimate of the effect of vitamin D on TB, especially in men. The participants in the control group was sampled from an urban population; but TB patients were from both urban and rural areas, as a result, the study's finding may not be generalizable to the rural population. Moreover, we did not systematically investigate active TB in the control group, and as a result, we could not exclude the possibility of inclusion of any TB cases in this group.

The finding of high prevalence of vitamin D insufficiency in TB patients has a number of clinical implications. Vitamin D in the form of cod liver oil and sunlight exposure was once a therapy for tuberculosis prior to the Robert Koch's discovery of the etiology of this disease. The association between vitamin D insufficiency and the risk of tuberculosis suggests that supplementation of vitamin D may help prevent and reduce the severity of tuberculosis. Indeed, a recent study [27] has shown that the severity of TB at the end of treatment was less for patients with normal vitamin D status at baseline than for those with vitamin D insufficiency, without adverse effects. However, the vitamin D dose used in the intervention (100,000 IU) is probably too low to warrant a clinical effect. These results taken together suggest that low vitamin D status in TB patients, whether cause or effect, might be an important determinant of treatment outcome and comorbidities.

## Conclusions

In summary, vitamin D insufficiency was associated with an increased risk of tuberculosis in Vietnam, but the association was observed in men, not in women. Considering findings from previous work, and given the current epidemics of vitamin D insufficiency in the world and in Vietnam, the present finding warrants further studies to determine whether vitamin D supplementation can have a role in the prevention and treatment of tuberculosis in developing countries.

## Competing interests

All authors declare that they do not have any conflict of interest with regarding to this work. Professor T. Nguyen received honorarium for speaking and providing consultant services to MSD Vietnam Ltd, Sanofi-Aventis, Norvatis, and Roche.

## Authors' contributions

LHP and TV conceived the study concept and designed the study; LHP, PKB and TTN carried out data collection; TTN carried out the biochemical analysis, including measurement of 25(OH)D; LHP and NDN performed the statistical analysis; LHP, NDN and TVN participated in the drafting of manuscript. All authors read and approved the final manuscript.

## Pre-publication history

The pre-publication history for this paper can be accessed here:

http://www.biomedcentral.com/1471-2334/10/306/prepub
